# Heat shock protein 90 in neurodegenerative diseases

**DOI:** 10.1186/1750-1326-5-24

**Published:** 2010-06-03

**Authors:** Wenjie Luo, Weilin Sun, Tony Taldone, Anna Rodina, Gabriela Chiosis

**Affiliations:** 1 Laboratory of Molecular and Cellular Neuroscience, The Rockefeller University and Fisher Foundation for Alzheimer's Disease, New York, NY 10021, USA; 2 Department of Medicine and Program in Molecular Pharmacology and Chemistry, Memorial Sloan-Kettering Cancer Center, New York, NY 10021, USA

## Abstract

Hsp90 is a molecular chaperone with important roles in regulating pathogenic transformation. In addition to its well-characterized functions in malignancy, recent evidence from several laboratories suggests a role for Hsp90 in maintaining the functional stability of neuronal proteins of aberrant capacity, whether mutated or over-activated, allowing and sustaining the accumulation of toxic aggregates. In addition, Hsp90 regulates the activity of the transcription factor heat shock factor-1 (HSF-1), the master regulator of the heat shock response, mechanism that cells use for protection when exposed to conditions of stress. These biological functions therefore propose Hsp90 inhibition as a dual therapeutic modality in neurodegenerative diseases. First, by suppressing aberrant neuronal activity, Hsp90 inhibitors may ameliorate protein aggregation and its associated toxicity. Second, by activation of HSF-1 and the subsequent induction of heat shock proteins, such as Hsp70, Hsp90 inhibitors may redirect neuronal aggregate formation, and protect against protein toxicity. This mini-review will summarize our current knowledge on Hsp90 in neurodegeneration and will focus on the potential beneficial application of Hsp90 inhibitors in neurodegenerative diseases.

## Roles of Hsp90 in neurodegeneration

Hsp90 is a molecular chaperone with important roles in maintaining the functional stability and viability of cells under a transforming pressure [1-3]. For neurodegenerative disorders associated with protein aggregation, the rationale has been that inhibition of Hsp90 activates heat shock factor-1 (HSF-1) to induce production of Hsp70 and Hsp40, as well as of other chaperones, which in turn, promote disaggregation and protein degradation [4-6]. However, recent evidence reveals an additional role for Hsp90 in neurodegeneration. Namely, Hsp90 maintains the functional stability of neuronal proteins of aberrant capacity, thus, allowing and sustaining the accumulation of toxic aggregates [7,8]. Below, we summarize the current understanding on these Hsp90 biological roles and review potential applications of pharmacological Hsp90 inhibition in neurodegenerative diseases.

### 1. HSF-1 is a master regulator of the heat shock response

**Figure 1:**
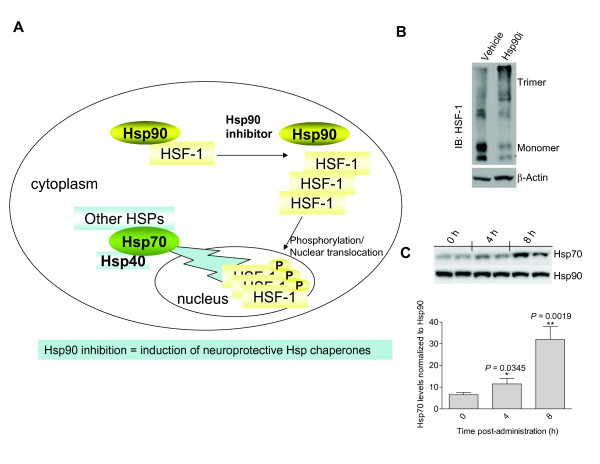
**Heat shock proteins are induced upon Hsp90 inhibition**. (**A**) Schematic representation of HSF-1 regulation by Hsp90 and its activation by Hsp90 inhibitors. (**B**) Treatment of cells with heat shock or an Hsp90 inhibitor (Hsp90i) results in HSF-1 trimerization [10]. (**C**) Systemic administration of the purine-scaffold Hsp90 inhibitor PU-DZ8 to AD transgenic mice results in Hsp70 induction in the brain [29].

Exposed to conditions of stress, cells normally respond by activation of the heat shock response (HSR) accompanied by increased synthesis of a number of cytoprotective heat shock proteins (Hsps) which dampen cytotoxicity, such as caused by misfolded and denatured proteins [4-6]. The most prominent part of this transition occurs on the transcriptional level. In mammals, protein-damaging stress is regulated by activation of HSF-1, which binds to upstream regulatory sequences in the promoters of heat shock genes [9]. The activation of HSF-1 proceeds through a multi-step pathway, involving a monomer-to-trimer transition, nuclear accumulation and extensive posttranslational modifications (Fig. (1A)). The function of HSF-1 is regulated by Hsp90 [10]. Namely, under non-stressed conditions, Hsp90 binds to HSF-1 and maintains the transcription factor in a monomeric state. Stress, heat shock or inhibition of Hsp90 release HSF-1 from the Hsp90 complex, which results in its trimerization (Fig. (1B)), activation and translocation to the nucleus where it initiates a heat shock response, manifested in the production of Hsps such as the chaperones Hsp70 and its activator, Hsp40 (Fig. (1A,C)). Neurons in the differentiated state, both *in vivo *and *in vitro *systems have been reported to be resistant to Hsp induction following conventional heat shock [5]. In contrast, pharmacologic induction of Hsp70 upon HSF-1 activation has been documented, and moreover as described below, demonstrated to be protective in neurons against toxicity caused by multiple types of insults.

#### 1.1. Hsp70 redirects neuronal aggregate formation and protects against aggregate toxicity

Frequently, neurodegenerative diseases are characterized by a gain of toxic function of misfolded proteins. Here, toxicity may result from an imbalance between normal chaperone capacity and production of dangerous protein species. Increased chaperone expression can suppress the neurotoxicity of these molecules, suggesting possible therapeutic strategies. Indeed, several studies summarized below, have reported a reduction in cellular toxicity upon expression of Hsp70 and Hsp40 in neurodegenerative aggregation disease models.

The polyglutamine (polyQ) diseases consist of nine neurodegenerative diseases in which a polyQ tract expansion leads to protein misfolding and subsequent deposition of protein aggregates in neurons [11]. Among these are Huntington's disease (HD), spinal and bulbar muscular atrophy (SBMA), Dentatorubral-pallidoluysian atrophy (DRPLA), and several ataxias (SCA1-3). In HD, mutant forms of huntingtin (htt) with expanded glutamine repeats form nuclear and cytoplasmic aggregates. Muchowski *et al *found that Hsp70 and its cochaperone Hsp40 suppressed the assembly of htt into spherical and annular polyglutamine oligomers and thus attenuated the formation of detergent-insoluble amyloid-like fibrils [12]. Likewise, in a yeast model of HD, expression of Hsp70 and Hsp40 reduced the toxicity associated with expression of mutant htt by preventing its aberrant interaction with an essential polyQ-containing transcription factor [13]. Studies in a mouse model of HD suggested that in neurons, protection by Hsp70 against the toxic effects of misfolded htt protein occurred by mechanisms independent of the deposition of fibrillar aggregates, namely by binding monomeric and/or low molecular mass SDS-soluble oligomers that are likely off-pathway to fibril formation, but may be potentially pathogenic [14]. In a mammalian model of spinocerebellar ataxia (SCA) type 1, expression of Hsp70 afforded protection against polyQ-induced neurodegeneration [15]. Pharmacological induction of heat-shock proteins in spinal and bulbar muscular atrophy (SBMA)-transgenic mice suppressed nuclear accumulation of the pathogenic androgen receptor (AR) protein, resulting in amelioration of polyglutamine-dependent neuromuscular phenotypes [16].

In amyotrophic lateral sclerosis (ALS), the FDA-approved drug riluzole was reported to partly act by HSF-1 activation and amplification of the HSR [17]. In the Superoxide dismutase 1 (SOD1) mouse model of ALS, elevation in levels of Hsp70 by another agent, arimoclomol, protected motor-neurons in both acute injury-induced motor-neuron degeneration as well as progressive motor-neuron degeneration models [18].

In various cellular models of Alzheimer's disease (AD), increased levels of Hsp70 promoted tau solubility and tau binding to microtubules [19]. Hsp70 also inhibited the propensity of Aβ to aggregate [20], and reduced the toxicity of Aβ on neuronal cultures [21]. Moreover, Amyloid precursor protein (APP) and/or its amyloidogenic derivative Aβ are targets of chaperone mediated clearance [22,23]. In *Drosophila melanogaster *and yeast models of Parkinson's disease (PD), directed expression of Hsp70 or pharmacologic Hsp modulation prevented the neuronal loss caused by α-synuclein [24,25]. Huang *et al *reported that these effects of Hsp70 manifested by inhibition of α-synuclein fibril formation via preventing the formation of prefibrillar α-synuclein formation [26]. Using α-synuclein deletion mutants, Luk *et al *indicated that interactions between the Hsp70 substrate binding domain and the α-synuclein core hydrophobic region mediated assembly inhibition, and that the assembly process was inhibited prior to the elongation stage [27].

Overall, in the several neurodegenerative disease models presented above, overexpression of Hsp70 improved the severities of several disease phenotypes without visibly affecting aggregate formation, suggesting that chaperones do not prevent aggregation *per se*, but rather redirect aggregates into amorphous deposits, thereby sequestering potentially toxic species from bulk solution.

#### 1.2. Expression of Hsp70 and other Hsps are induced upon pharmacologic Hsp90 inhibition

**Figure 2:**
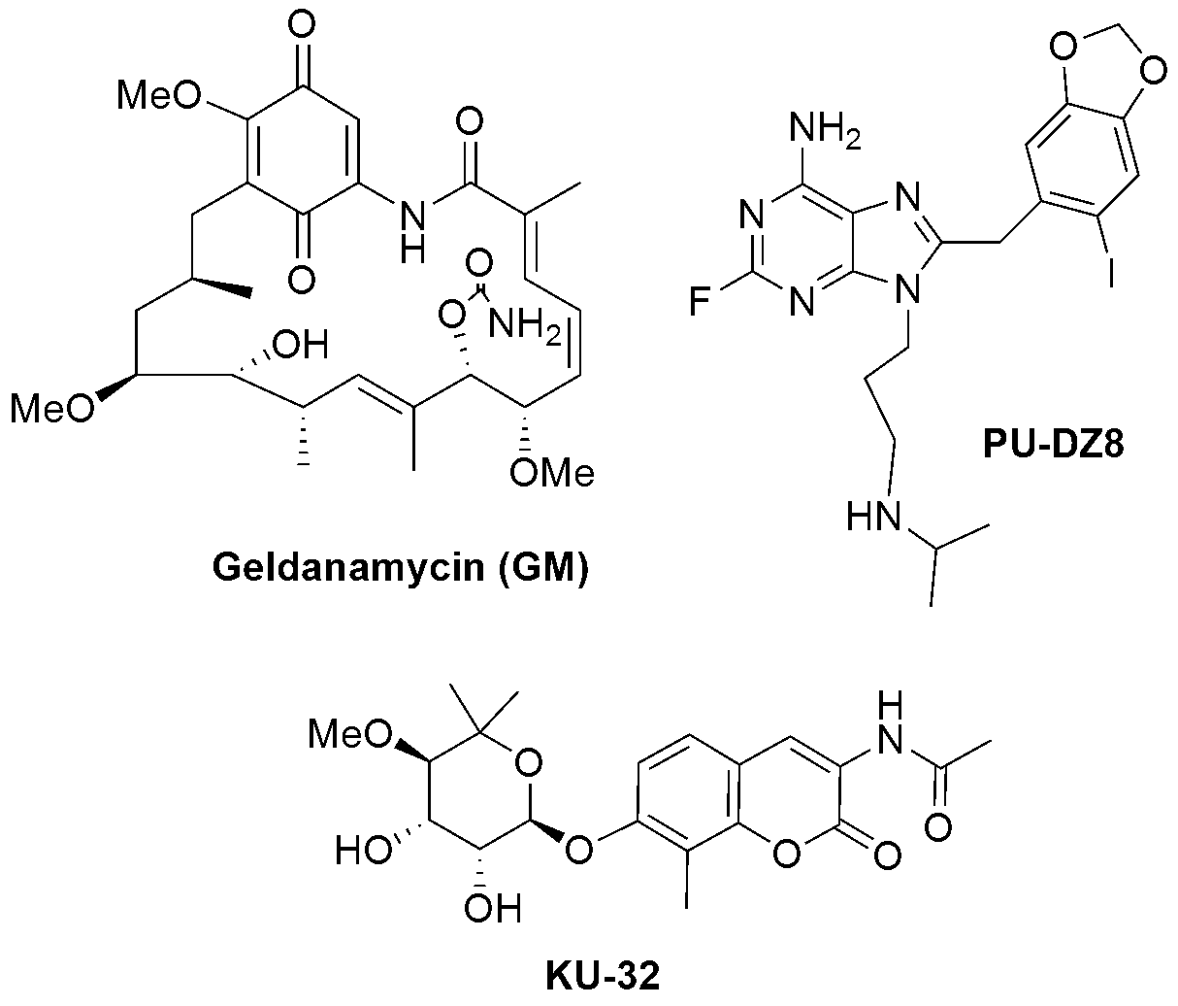
**Chemical structures of several representative Hsp90 inhibitors**. GM = ansamycin class; PU-DZ8 = purine-scaffold class; KU-32 = novobiocin class.

As noted above, inhibition of Hsp90 releases HSF-1 from the Hsp90 complex resulting in subsequent production of Hsps (Fig. (1)), and induction of Hsp70 by Hsp90 inhibitors is well documented in neurodegenerative disease models. Geldanamycin (GM) (Fig. (2)), an Hsp90 inhibitor [28], induced a dose-dependent increase of Hsp70 in an AD cell model, as well as in rat primary cortical neurons [19] and reduced the amount of insoluble tau and the basal levels of okadaic-acid induced tau phosphorylation [19]. Treatment of primary cortical neurons with the purine-scaffold Hsp90 inhibitor PU24FCl, led to a dose-dependent increase in Hsp70 [29]. Similarly, administration of two CNS-permeable PU24FCl-derivatives, PU-DZ8 (Fig. (2)) [29] and EC102 [30], to tau transgenic mice (htau and JNPL3) resulted in Hsp70 induction in the brain, effects maintained at 24 h post-administration. KU32, an Hsp90 inhibitor of distinct chemical nature (Fig. (2)), induced Hsp70 in SH-SY5Y neuroblastoma cultures and protected them against Aβ-induced toxicity [31]. GM activated a heat shock response and inhibited htt aggregation in a cell culture model of HD [32,33] and induced Hsp70 in a time- and concentration-dependent manner and prevented α-synuclein aggregation and protected against toxicity in a cellular α-synuclein aggregation model [34]. Auluck *et al *reported that treatment of a fly model of PD with GM fully protected against α-synuclein toxicity [35]. GM also protected against 1-methyl-4-pheny-1,2,3,6-tetrahydropyridine (MPTP)-induced dopaminergic neurotoxicity, a mouse model of PD [36]. Pretreatment with GM via intracerebral ventricular injection, prior to MPTP treatment, induced Hsp70 and increased residual dopamine content and tyrosine hydroxylase immunoreactivity in the striatum [36]. Hsp70 induction in the spinal cord was noted upon intraperitoneal injection of a GM derivative, 17-AAG, in a mouse model of SBMA [37]. 17-AAG was also effective against neurodegeneration in other polyQ diseases [38]. Namely, it suppressed compound eye degeneration and inclusion body formation and rescued the lethality in a *Drosophila *model of SCA. It also suppressed neurodegeneration in a HD fly model. Knockdown of HSF-1 abolished the induction of molecular chaperones and the therapeutic effect of 17-AAG on polyQ-induced neurodegeneration in the *Drosophila *models, arguing that the therapeutic effect of 17-AAG was mainly HSF1-mediated [38].

In summary, HSF-1 activation by Hsp90 inhibitors was noted in several *in vitro* and *in vivo* models of neurodegenerative disease, suggesting Hsp90 inhibition as a means to modulate Hsp levels in the diseased brain, with the goal of protecting against the toxic proteins that arise during the neurodegenerative process.

### 2. Inhibition of Hsp90 reduces aberrant neuronal protein activity and expression

**Figure 3:**
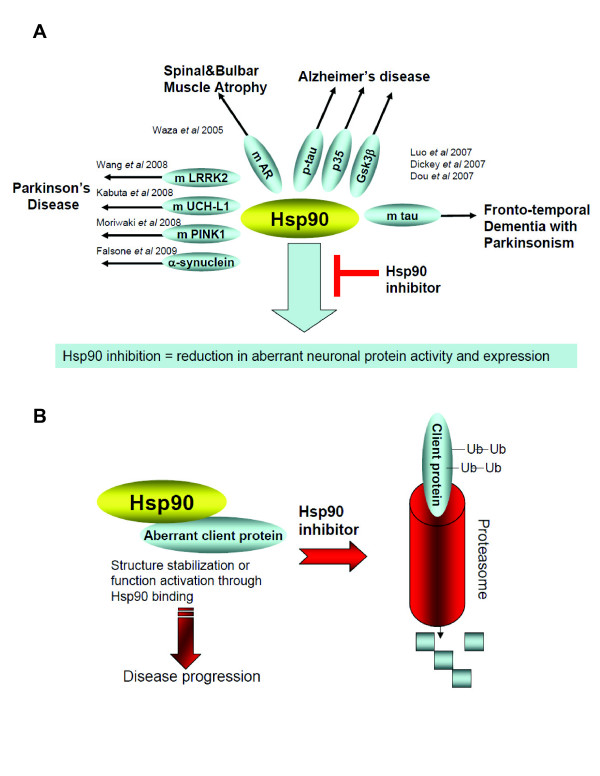
**Hsp90 shelters aberrant neuronal proteins**. **(A) **Aberrant neuronal proteins regulated by Hsp90. To tolerate the accumulation of dysregulated processes and to allow the development of the disease phenotype, the functional stability of these aberrant processes likely requires a "buffering" mechanism, such as offered by Hsp90. These aberrant neuronal proteins activities develop Hsp90-dependency and promote disease progression. (**B**) Pharmacologic Hsp90 inhibition results in inactivation or degradation of Hsp90-regulated proteins, mainly by a proteasomal pathway.

In addition to regulation of HSF-1, recent evidence suggests an additional role for Hsp90 in maintaining the functional stability of neuronal proteins of aberrant capacity (Fig. (3A)).

SBMA is an inherited motor neuron disease caused by the expansion of a polyglutamine tract within the androgen receptor (AR) [11]. The pathologic features of SBMA are motor neuron loss in the spinal cord and brainstem and diffuse nuclear accumulation and nuclear inclusions of the mutant AR (mAR) in the residual motor neurons and certain visceral organs. Waza *et al *demonstrated that mAR present in SBMA is one of the proteins regulated by Hsp90 (Fig. (3A)) [37]. Hsp90 formed a molecular complex with mAR to maintain its functional stability. In both SBMA cell models and transgenic mice, inhibition of Hsp90 by 17-AAG led to a preferential degradation of the mAR, mainly by the proteasome machinery. These effects of 17-AAG were uncoupled from induction of Hsp70, and resulted from direct destabilization of mAR and its subsequent degradation upon Hsp90 inhibition. In a transgenic mouse model of SBMA, 17-AAG ameliorated motor impairments without detectable toxicity, and reduced the amounts of monomeric and aggregated mAR [37]. Similar findings were reported by Thomas *et al*; these authors found that pharmacologic Hsp90 inhibition blocked the development of aggregates of the expanded glutamine androgen receptor (AR112Q) in HSF1(-/-) mouse embryonic fibroblasts where Hsp70 and Hsp40 chaperones were not induced [39].

Parkinson disease (PD), the most common neurodegenerative movement disorder, is characterized by a complexity of pathogenic events [40], many of which were recently linked to Hsp90 (Fig. (3A)). Wang *et al *have recently shown that Leucine-rich repeat kinase 2 (LRRK2), a kinase of whose mutated forms is prevalent in both familial and apparently sporadic cases of PD, formed a complex with Hsp90 *in vivo *[41]. Inhibition of Hsp90 function by the purine-scaffold Hsp90 inhibitor PU-H71 disrupted the association of Hsp90 with LRRK2 and led to elimination of LRRK2 by the proteasome. PU-H71 limited the mutant LRRK2-elicited toxicity to neurons and rescued the axon growth retardation defect caused by the LRRK2 G2019S mutation in neurons [41]. Mutation of PTEN-induced kinase 1 (PINK1), which encodes a putative mitochondrial serine/threonine kinase, leads to PARK6, an autosomal recessive form of familial Parkinson's disease [40]. The recessive inheritance of this form of Parkinson's disease suggests loss of PINK1 function is closely associated with its pathogenesis. Moriwaki *et al *have reported that Hsp90 binds PINK1 to enhance its stability. In cells treated with the Hsp90 inhibitor GM, levels of PINK1 were greatly diminished via the ubiquitin-proteasome pathway [42]. α-Synuclein is an intrinsically unstructured protein that may form fibrils, and is also involved in PD neurodegeneration [40]. Falsone *et al *has recently reported that Hsp90 influences α-synuclein vesicle binding and amyloid fibril formation, two processes that are tightly coupled to α-synuclein folding [43]. Namely, Hsp90 bound α-synuclein and abolished the interaction of this polypeptide with small unilamellar vesicles. Hsp90 also promoted fibril formation in an ATP-dependent manner via oligomeric intermediates [43]. Another link between α-synuclein and Hsp90 was provided by Kabuta *et al *[44]. Alpha-synuclein is degraded at least partly by chaperone-mediated autophagy (CMA). The authors suggested that aberrant interaction of mutant ubiquitin C-terminal hydrolase L1 (UCH-L1) with the chaperone-mediated autophagy CMA machinery, at least partly accounted for the pathogenesis of PD associated with I93M UCH-L1 and the increase in the amount of α-synuclein [44].

In tauopathies, neurodegenerative diseases characterized by tau protein abnormalities, transformation is characterized by abnormalities in the tau protein leading to an accumulation of hyperphosphorylated and aggregated tau [45]. In AD, tau hyperphosphorylation is suggested to be a pathogenic process caused by aberrant activation of several kinases, in particular cyclin-dependent protein kinase 5 (CDK5) and glycogen synthase kinase-3 beta (GSK3β), leading to phosphorylation of tau on pathogenic sites [46]. Hyperphosphorylated tau in AD is believed to misfold, undergo net dissociation from microtubules and form toxic tau aggregates. In a cluster of tauopathies termed "frontotemporal dementia and parkinsonism linked to chromosome 17 (FTDP-17)", transformation is caused by several mutations in human tau isoforms on chromosome 17, that result in and are characterized by the accumulation of aggregated tau similar to that in AD [47]. Luo *et al *have reported that the stability of p35 and p25, neuronal proteins that activate CDK5 through complex formation leading to aberrant tau phosphorylation, and that of mutant but not wild type tau protein, were maintained in tauopathies by Hsp90 (Fig. (3A)) [29]. Inhibition of Hsp90 in both cellular and mouse models of tauopathies led to reduction of the pathogenic activity of these proteins and resulted in a dose- and time-dependent elimination of aggregated tau [29]. When administered 5xweek for 30 days to JNPL3 transgenic (tg) mice, PU-DZ8 (Fig. (2), led to significant reduction in mutant tau expression and phosphorylation without toxicity to the mice [29]. Complementarily, Dickey *et al *demonstrated that the EC102 Hsp90 inhibitor promoted selective decrease in ptau species in a tg mouse model of AD independent of HSF-1 activation (Fig. (3A)) [30]. Both reports identify the proteasomal pathway as responsible for degradation of the aberrant tau species following Hsp90 inhibition [29,30]. A link between Hsp90 and GSK3β was reported by Dou *et al *(Fig. (3A)) [48]. Namely, the stability and function of the GSK3β was found to be maintained by Hsp90, and Hsp90 inhibition by GM and PU24FCl led to a reduction in the protein level of GSK3β, effect associated with a decrease in tau phosphorylation at putative GSK3β sites [48]. Further, Tortosa *et al *reported that binding of Hsp90 to tau facilitates a conformational change in tau that could result in its phosphorylation by GSK3 and its aggregation into filamentous structures [49].

Collectively, the above data suggest that at the phenotypic level, Hsp90 appears to serve as a biochemical buffer for the numerous aberrant processes that facilitate the evolution of the neurodegenerative phenotype (Fig. (3A)). Inhibition of Hsp90 by small molecules results in the destabilization of the Hsp90/aberrant protein complexes leading primarily to degradation of these proteins by a proteasome-mediated pathway (Fig. (3B)).

## Conclusion

Collectively, these reports suggest that in neurodegenerative diseases Hsp90 inhibition may offers a dual therapeutic approach. On one hand, its benefits may come from induction of Hsp70 and other chaperones able of redirecting neuronal aggregate formation, and capable of protective potential against protein toxicity, proposing Hsp90 inhibition as a pharmacological intervention to therapeutically increase expression of molecular chaperone proteins to treat neurodegenerative diseases where aggregation is central to the pathogenesis (Fig. (1)). On the other hand, Hsp90 inhibition may ameliorate protein hyperphosphorylation and subsequent aggregation by reduction of aberrant neuronal protein activity (Fig. (3)). The usefulness of Hsp90 inhibitors as clinical agents in neurodegenerative diseases will depend on whether their effects occur at concentrations of drug that are not toxic and on whether the drugs can be administered chronically in such a fashion so as to safely achieve these concentrations in the brain. While studies in several cellular models show promise for this class of compounds in treating a large spectrum of neurodegenerative diseases, these studies need to be furthered in animal models, with the goal of testing both Hsp90 inhibitors efficacy in improving neuro-pathology and their safety under long-term administration schedules. While several of the studies have used GM and its derivatives, these agents have several liabilities that limit their future clinical use [50]. Development for cancers of Hsp90 inhibitors of scaffolds distinct from that of GM is currently reaching an explosive phase, where several agents are in clinical evaluation, with many others following behind [50]. It is likely that the Hsp90 inhibitor classes with best safety profiles will also move into the neurodegenerative space. It now remains the goal of medicinal chemistry to deliver CNS-permeable Hsp90 inhibitors with a good therapeutic index to fulfill the promise of these agents in the treatment of neurodegenerative diseases.

## List of abbreviations

AD: Alzheimer's disease; APP: amyloid precursor protein; ALS: amyotrophic lateral sclerosis; AR: androgen receptor; CDK5: cyclin-dependent protein kinase 5; CMA: chaperone-mediated autophagy; CNS: central nervous system; DRPLA: Dentatorubral-pallidoluysian atrophy; FTDP-17: frontotemporal dementia and parkinsonism linked to chromosome 17; FDA: Food and Drug Administration; GM: Geldanamycin; GSK3β: glycogen synthase kinase-3 beta; HD: Huntington's disease; HSF-1: heat shock factor-1; HSR: heat shock response; Hsps: heat shock proteins; Hsp90: Heat shock protein 90; Hsp70: Heat shock protein 70; Hsp90i: Hsp90 inhibitor; Htt: huntingtin; LRRK2: Leucine-rich repeat kinase 2; MPTP: 1-methyl-4-pheny-1,2,3,6-tetrahydropyridine; PARK6: Parkinson disease 6: autosomal recessive early-onset; PD: Parkinson disease; PolyQ: polyglutamine diseases; PINK1: PTEN-induced kinase 1; SBMA: spinal and bulbar muscular atrophy; SCA: spinocerebellar ataxia; SDS: sodium dodecyl sulfate; SOD1: Superoxide dismutase 1; Tg: transgenic; UCH-L1: ubiquitin C-terminal hydrolase L1;

## Competing interests

The authors are inventors on patents and patent applications relating to Hsp90 compositions of matter and to the use oh Hsp90 inhibitors in neurodegenerative diseases.

## Authors' contributions

All authors contributed to the concept and writing the review article, and read and approved the final manuscript.
